# Correlation between LTC4S -444 A>C polymorphism and susceptibility to asthma: A meta-analysis and trial sequential analysis

**DOI:** 10.5937/jomb0-44538

**Published:** 2024-01-25

**Authors:** Delin Wu, Yuna Liu, Yan Liu, Najuan Cui, Yan Zhu, Sidao Zheng, Shaohua Wang

**Affiliations:** 1 Beijing Hospital of Integrated Traditional Chinese and Western Medicine, Department of Respiratory, Beijing, China; 2 Beijing Hospital of Integrated Traditional Chinese and Western Medicine, Department of Science & education, Beijing, China; 3 Beijing Hospital of Integrated Traditional Chinese and Western Medicine, Department of Cardiology, Beijing, China

**Keywords:** LTC4S -444 A>C polymorphism, susceptibility, asthma, meta-analysis, LTC4S -444 A>C polimorfizam, osjetljivost, astma, meta-analiza

## Abstract

**Background:**

This study aims to uncover the potential correlation between LTC4S -444 A>C polymorphism and susceptibility to asthma.

**Methods:**

Literatures reporting the correlation between LTC4S -444 A>C polymorphism and susceptibility to asthma published before 1st June, 2019 were searched in PubMed, Embase, Cochrane, Wanfang and CNKI. Eligible literatures were enrolled and their data were extracted. OR and its 95% CI were calculated for assessing the correlation between LTC4S -444 A>C polymorphism and susceptibility to asthma. The included data were weighted by an inverse variance and then analyzed by a fixed or random effects model. Heterogeneity test and sensitivity analysis were performed on the enrolled reports. STATA12.1 and TSA (trial sequential analysis) were utilized for analyses.

## Abstract (sr)

**Uvod:**

Ova studija ima za cilj da otkrije potencijalnu korelaciju između LTC4S -444 A>C polimorfizma i podložnosti astmi.

**Metode:**

Literatura koja izveštava o korelaciji između LTC4S -444 A>C polimorfizma i podložnosti astmi objavljena pre 1. juna 2019. pretražena je u PubMed, Embase, Cochrane, Vanfang i CNKI. Prihvatljiva literatura je upisana i njihovi podaci su izvučeni. OR i njegov 95% CI su izračunati za procenu korelacije između LTC4S -444 A>C polimorfizma i podložnosti astmi. Uključeni podaci su ponderisani inverznom varijansom, a zatim analizirani pomoću modela fiksnih ili slučajnih efekata. Ispitivanje heterogenosti i ana liza osetljivosti urađeni su na upisanim izveštajima. Za analize su korišćeni STATA12.1 i TSA (probna sekvencijalna analiza).

**Rezultati:**

Uključeno je 15 studija koje su uključivale 3.791 pacijenta sa astmom i 2.185 zdravih kontrola. Nije pronađena značajna korelacija između polimorfizma LTC4S - 444 A>C i podložnosti astmi prema rezultatima različitih modela ((Dominantni model (D): OR=1,10, 95% CI=0,98 -1,23; Recesivni model (R)): 1,07, 0,84-1,36; Homozigotni model (Homo): 1,11, 0,87-1,41; Heterozigotni model (Hetero): 1,10, 0,98-1,24; Alel model (A): 1,07, 0,98-1. Analize sprovedene u Aziji i bele populacije, kao i u populacijskim i bolničkim kontrolama dobili su iste zaključke.

**Zaključak:**

Nije identifikovana značajna korelacija između LTC4S -444 A>C polimorfizma i podložnosti astmi. Za dalju validaciju u multicentričnoj bolnici potrebna su istraživanja visokog kvaliteta i velike veličine uzorka.

## Introduction

Bronchial asthma is a chronic allergic condition of the respiratory tract. The pathogenesis of asthma is complex, involving diverse inflammatory cells and structural cells [1] [2] [3]. Asthma-induced chronic inflammation and structural change result in the high reactivity of the airways and limitation of the generally reversible expiratory flow [4] [5]. It is estimated that there are approximately 300 million people suffering from asthma and 180,000 people die of asthma globally [6] [7]. The etiology and pathogenesis of asthma have not been comprehensively explored yet [8] [9]. Generally, environmental and genetic factors both contribute to the occurrence of asthma [10]. Exposure to allergic substances, pollutants, tobacco and smog have been recognized as intrinsic and extrinsic risk factors for asthma [11]. Individuals with different genetic backgrounds exhibit different levels of susceptibility to asthma, highlighting the role of genetic components in the occurrence and progression of asthma [12] [13].

LTC4S is a key enzyme for the production of cysteinyl leukotrienes (CysLTs). Polymorphism A-444C of Cytidine (C) instead of Adenosine (A) is present in promoter region -444 of LTC4S gene. The frequency of C (-444) variant allele is 22.6% in normal controls and 43.6% in aspirin asthma patients [14] [15]. Relevant studies have uncovered that LTC4S is upregulated in eosinophils of individuals carrying C-444 allele, thus contributing to the enhanced intracellular synthesis capacity of CysLTs [16] [17]. Current researches focus on genetic understanding of asthma pathogenesis [17]. It is reported that LTC4S -444 A>C polymorphism in Han population is closely linked to disease severity and pulmonary dysfunctional level in adult non-acute asthma [18]. This polymorphism is identified to be closely related to high reactivity of the respiratory tract, chronic inflammation, respiratory remodeling, and decreased lung function in patients with bronchial asthma [15] [16] [17] [18].

So far, several researches on underlying the correlation between LTC4S -444 A>C polymorphism and susceptibility to asthma have been published [15] [16] [17] [18]. However, the conclusion was controversial. This study searched for relevant studies and analyzed their potential correlation.

## Materials and methods

### Literature search

Literatures reporting the correlation between LTC4S -444 A>C polymorphism and susceptibility to asthma published before 1^st^ June, 2019 were searched in PubMed, Embase, Cochrane, Wanfang and CNKI. Key words searched were as follows: »Leukotrienes C4 synthase« or »LTC4S -444 A>C« or »single nucleotide polymorphism« or »variants«, or »polymorphism«, and »asthma«, and »risk« or »susceptibility«. No limitations were set on publication regions. Enrolled studies and their citations were manually examined by two researchers independently. Studies with larger sample size or latest published were selected if data overlapping.

### Inclusion and exclusion criteria

Inclusion criteria were applied as follows: (1) Case-control or cohort studies; (2) Studies that analyzed the correlation between LTC4S -444 A>C polymorphism and susceptibility to asthma; (3) OR and 95% CI or relative data that could be used to calculate them were provided.

Exclusion criteria were applied as follows: (1) Cross-sectional studies, case reports, abstracts and reviews; (2) Studies that only analyzed asthma; (3) Inadequate data that could not calculate OR and their 95% CI; (4) Low-quality and repeated studies.

### Data extraction

Baseline data extraction: First author, year of research, ethnicity, control resource, genotyping method, OR and its 95% CI. Data acquisition was independently carried out by two reviewers, and a third reviewer was responsible for re-evaluating disagreements.

### Statistical analysis

The heterogeneity in enrolled studies was tested using the χ^2^ test at a test level of α= 0.10, and represented as *I* ^2^ value. Gene polymorphisms included in this analysis were studied in at least three case-control studies. P<0.10 or *I* ^2^ >50% was considered to be statistically heterogeneous and a random effects model was applied; Otherwise, a fixed effects model was adopted. OR and its 95% CI in each model were calculated and analyzed by Z test: D (AC + CC *vs*. AA); R (CC *vs*. AC + AA); (C) Homo (CC *vs.* AA); (D) Hetero (AC *vs*. AA); (E) A (C allele *vs*. A allele). Genotype in control group was calculated by χ^2^ test. P<0.05 considered that genotype in control group was not consistent with Hardy-Weinberg equilibrium (HWE). At last, Begg's test and Egger's test were utilized for evaluating publication bias. Statistical analysis was performed using Stata 12.1 and TSA.

## Results

### Characteristics of the studies

**Figure 1:**
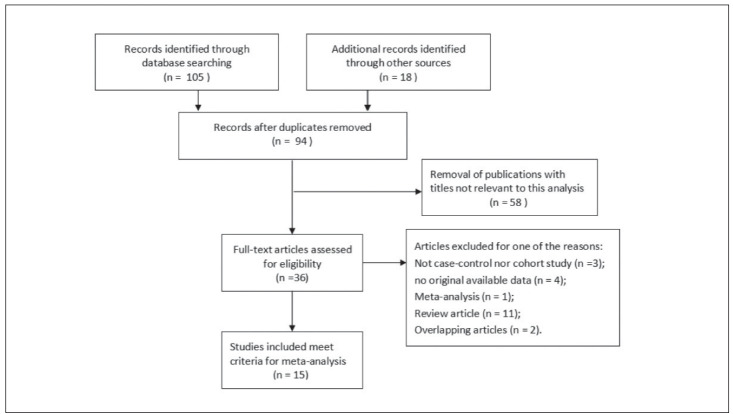
Flow diagram of literature search and selection process.

Fifteen studies involving 3,791 asthma patients and 2,185 healthy controls were enrolled [16] [18] [19] [20] [21] [22] [23] [24] [25] [26] [27] [28] [29] [30] [31]. Their baseline characteristics and genotype distribution were listed in Table 1. Flow diagram of literature search and selection process was depicted in Figure 1. Among the fifteen studies, 8 were carried out in Caucasian population, 6 were in Asian population and 1 was in African population. Besides, 4 studies were population-based and 11 were hospitalbased. Genotyping methods included sequenced, SNP-ITTM and PCR-RFLP.

**Table 1:** Characteristics of studies that investigated the association between LTC4S –444 A>C polymorphism and susceptibility to asthma. SOC: Source of controls; PB: Population-based controls; HB: Hospital-based controls; HWE: Hardy-Weinberg equilibrium.

Author	Year	Country	Ethnicity	SOC	Genotyping methods	No. of case	No. of control	Case (N)			Control (N)			HWE	II
								AA	AC	CC	AA	AC	CC		
Berghea	2015	Romania	Caucasian	PB	PCR-RFLP	104	103	54	38	12	60	34	9	Y	
Kang	2011	Korea	Asian	HB	PCR-RFLP	864	263	583	261	20	176	81	6	Y	
Xie	2010	China	Asian	HB	PCR-LDR	72	95	46	23	3	67	26	2	Y	12
Torres-Galvan	2009	Spain	Caucasian	HB	PCR-RFLP	110	82	65	40	5	45	33	4	Y	
Wu	2008	China	Asian	HB	PCR-RFLP	145	146	106	34	5	112	30	4	Y	8
Sanz	2006	Spain	Caucasian	HB	Sequenced	130	78	63	56	11	38	33	7	Y	27
Pan	2006	China	Asian	HB	PCR-RFLP	101	105	70	29	2	69	32	4	Y	
Choi	2006	Korea	Asian	HB	SNP-ITTM	234	124	164	68	2	94	26	4	Y	
Moissidis	2005	USA	African	PB	PCR-RFLP	30	60	26	4	0	54	6	0	Y	
Isidoro-Garcia	2005	Spain	Caucasian	HB	PCR-RFLP	123	103	58	54	11	55	41	7	Y	
Kedda	2004	Australia	Caucasian	HB	Sequenced	604	462	290	266	48	256	174	32	Y	
Sayers	2003	New	Caucasian	PB	PCR-RFLP	645	180	330	256	59	85	79	16	Y	
Asano	2002	Japan	Asian	HB	PCR-RFLP	349	171	225	113	11	107	54	10	Y	
Van Sambeek	2000	USA	Caucasian	HB	PCR-RFLP	94	137	50	32	12	73	53	11	Y	10
Sanak	2000	Poland	Caucasian	PB	PCR-RFLP	186	76	82	90	14	39	33	4	Y	

### Quantitative synthesis results

**Figure 2:**
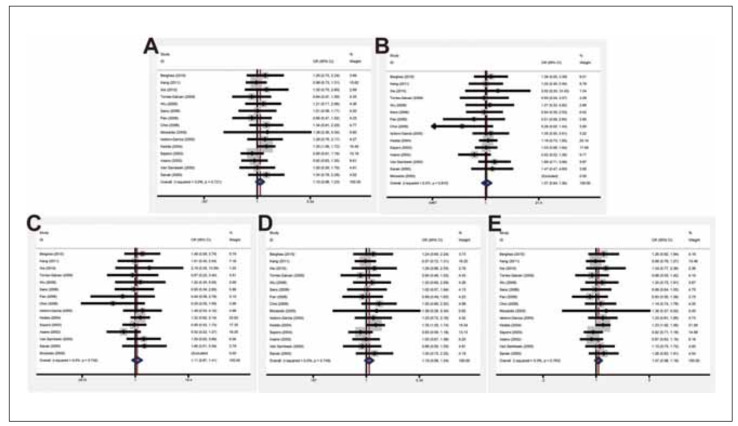
Forest plots of the correlation between LTC4S -444 A>C polymorphism and susceptibility to asthma in fixed-effects model.

No significant correlation between the LTC4S -444 A>C polymorphism and susceptibility to asthma was discovered according to the results of different models (D: OR=1.10, 95% CI=0.98-1.23; R: 1.07, 0.84-1.36; Homo: 1.11, 0.87-1.41; Hetero: 1.10, 0.98-1.24; A: 1.07, 0.98-1.18) (Table 2 and Figure 2).

**Table 2:** Meta-analysis results for the included studies of the association between LTC4S –444 A>C polymorphism and susceptibility to asthma.

Variables	No. of studies	Dominant model			Recessive model			Homozygous model			Heterozygous model			Allele model		
		OR<br>(95% CI)	P-values	I- squared (%)	OR<br>(95% CI)	P-values	I- squared (%)	OR<br>(95% CI)	P-values	I- squared (%)	OR<br>(95% CI)	p-values	I- squared (%)	OR<br>(95% CI)	P- values	I- squared (%)
–444 A>C		(AC + CC) vs. AA			CC vs. (AC + AA)			CC vs. AA			AC vs. AA			C vs. A		
All	15	1.10 (0.98–1.23)	0.721	0.0	1.07 (0.84–1.36)	0.810	0.0	1.11 (0.87–1.41)	0.742	0.0	1.10 (0.98–1.24)	0.749	0.0	1.07 (0.98–1.18)	0.763	0.0
Ethnicity																
Asian	6	1.04 (0.87–1.24)	0.705	0.0	0.75 (0.46–1.22)	0.483	0.0	0.76 (0.47–1.24)	0.487	0.0	1.07 (0.89–1.29)	0.711	0.0	1.00 (0.86–1.17)	0.658	0.0
Caucasian	8	1.14 (0.98–1.31)	0.439	0.0	1.19 (0.91–1.56)	0.983	0.0	1.24 (0.94–1.65)	0.950	0.0	1.11 (0.96–1.30)	0.426	0.3	1.12 (0.99–1.25)	0.611	0.0
Source of control																
HB	11	1.11 (0.98–1.27)	0.696	0.0	1.03 (0.78–1.37)	0.642	0.0	1.08 (0.81–1.44)	0.599	0.0	1.12 (0.98–1.28)	0.721	0.0	1.08 (0.97–1.20)	0.699	0.0
PB	4	1.04 (0.81–1.33)	0.395	0.0	1.17 (0.74–1.83)	0.804	0.0	1.16 (0.73–1.86)	0.592	0.0	1.01 (0.78–1.31)	0.452	0.0	1.05 (0.87–1.28)	0.450	0.0

**Figure 3:**
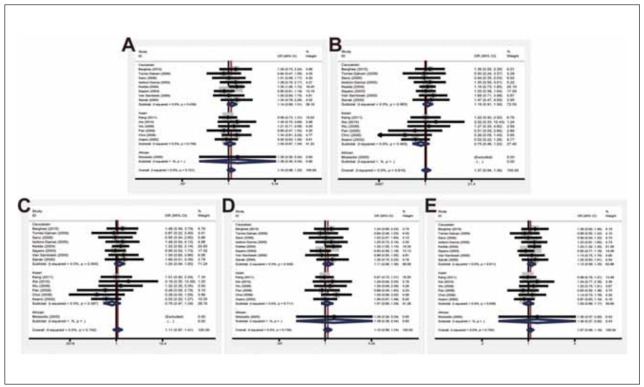
Forest plots of subgroup analysis by ethnicity of the correlation between LTC4S -444 A>C polymorphism and susceptibility to asthma in fixed-effects model. (A) Dominant model; (B) Recessive model; (C) Homozygous model; (D) Heterozygous model; (E) Allele model.

Subgroup analyses carried out in Asian (D: OR=1.04, 95% CI=0.87-1.24; R: 0.75, 0.46-1.22; Homo: 0.76, 0.47-1.24; Hetero: 1.07, 0.89-1.29; A: 1.00, 0.86-1.17) and Caucasian population (D: OR=1.14, 95% CI=0.98-1.31; R: 1.19, 0.91-1.56; Homo: 1.24, 0.94-1.65; Hetero: 1.11, 0.96-1.30; A: 1.12, 0.99-1.25) obtained the same findings (Figure 3).

**Figure 4:**
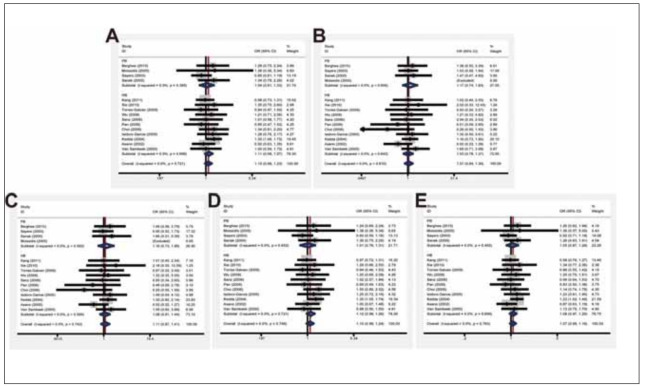
Forest plots of subgroup analysis by source of controls of the correlation between LTC4S -444 A>C polymorphism and susceptibility to asthma in fixed-effects model. (A) Dominant model; (B) Recessive model; (C) Homozygous model; (D) Heterozygous model; (E) Allele model.

In population-based (D: OR=1.04, 95% CI= 0.81-1.33; R: 1.17, 0.74-1.83; Homo: 1.16, 0.73-1.86; Hetero: 1.01, 0.78-1.31; A: 1.05, 0.87-1.28) and hospital-based (D: OR=1.11, 95% CI=0.98-1.27; R: 1.03, 0.78-1.37; Homo: 1.08, 0.81-1.44; Hetero: 1.12, 0.98-1.28; A: 1.08, 0.97-1.20) subjects, no significant relationship between the LTC4S -444 A>C polymorphism and susceptibility to asthma was observed as well (Figure 4).

### Heterogeneity

**Figure 5:**
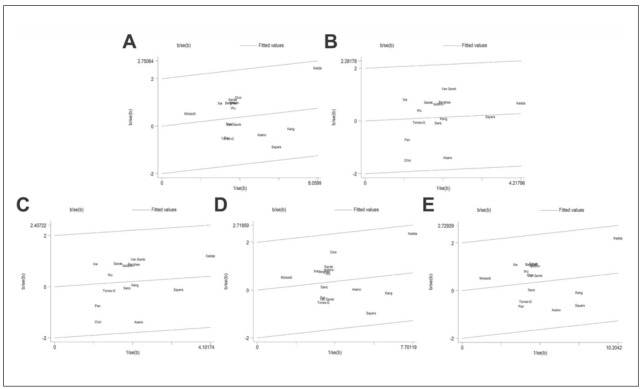
Galbraith plot of the correlation between LTC4S -444 A>C polymorphism and susceptibility to asthma. (A) Dominant model; (B) Recessive model; (C) Homozygous model; (D) Heterozygous model; (E) Allele model.

Heterogeneity was observed in all genetic models. Interestingly, subgroup analysis can reduce heterogeneity. In this analysis, neither the ethnicity nor control source can lead to heterogeneity. Galbraith radial plots in five genetic models showed no significant heterogeneity (Figure 5).

### Sensitivity analysis

**Figure 6:**
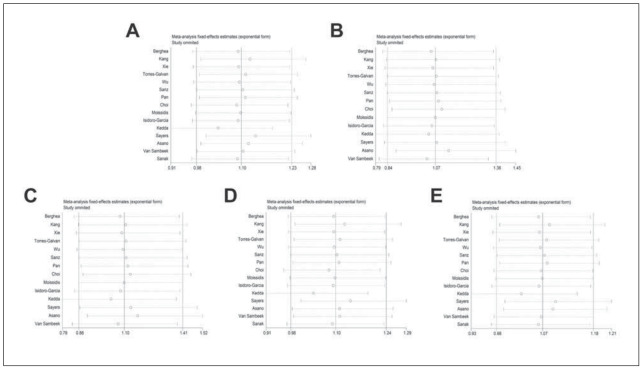
Sensitivity analysis in fixed model. (A) Dominant model; (B) Recessive model; (C) Homozygous model; (D) Heterozygous model; (E) Allele model.

Individual influence on OR was assessed by sensitivity analysis. Pooled OR in our analysis was not influenced by removal of any single research each time, verifying the robust conclusion (Figure 6).

### Publication bias

**Figure 7:**
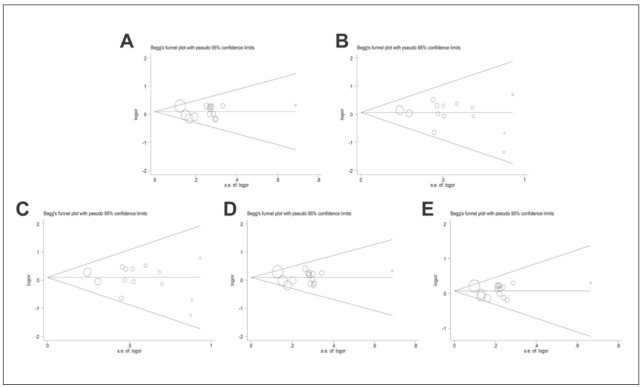
Begg’s funnel plot of publication bias test. (A) Dominant model; (B) Recessive model; (C) Homozygous model; (D) Heterozygous model; (E) Allele model.

Publication bias in this study was assessed using Begg's test and Egger's test. The systematic shape of funnel diagram indicated no significant publication bias (D: P=0.882; R: P=0.547; Homo: P=0.412; Hetero: P=0.805; A: P=0.729) (Figure 7). 

### TSA results

**Figure 8:**
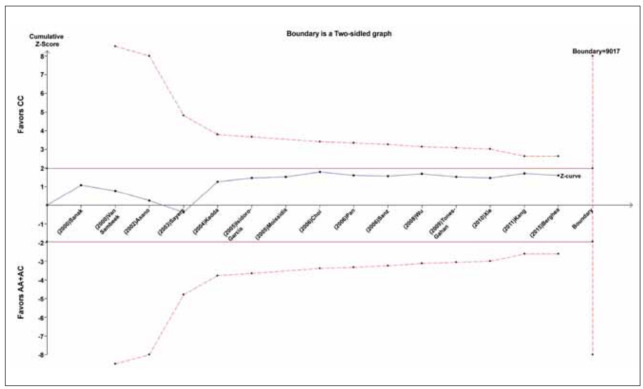
Trial sequential analysis of the correlation between LTC4S -444 A>C polymorphism and susceptibility to asthma. The required information size was calculated based on a two side α= 5%, β= 15% (power 85%), and a relative risk reduction of 20%.

The cumulative z-curve did not cross the test sequence monitoring boundary. Meanwhile, case numbers did not exceed the required amount of information, indicating that our conclusion are required for further conclusive evidence (Figure 8).

## Discussion

Bronchial asthma is a common chronic respiratory disease. Its morbidity and mortality throughout the world have been risen sharply [1] [2] [3]. It is estimated that there are over 25 million asthma patients in China, most of whom are children [4] [5]. Prevention and intervention of asthma are insufficient in our country [5] [6] [7]. In recent years, genetic mutations are considered to be important risk factors for asthma. A great number of susceptible genes to asthma have been identified [9] [10] [11]. Researches on candidate genes associated with susceptibility to bronchial asthma have become a hot topic in etiology [12] [13].

LTC4S is an important enzyme in the cysteine leukotriene synthesis pathway. As a strong inflammatory mediator, cysteinyl leukotriene is widely involved in many inflammatory pathological processes [14] [15]. Cysteinyl leukotrienes is able to alter endothelial cell permeability and vascular endothelial cell migration by activating their receptors CysTL1 and CysTL2, thus influencing smooth muscle spasm and microvascular leakage. LTC4S is located on the chromosome 5q35 [16] [17] [18]. Current researches on the correlation between LTC4S -444 A>C and asthma are controversial [17] [18].

Meta-analysis is a powerful tool that yields a more credible conclusion than that of an individual study, especially in controversial conclusions obtained from one common research [20] [21] [22] [23] [24] [25] [26] [27] [28] [29] [30] [31]. In this paper, 15 independent case-control studies involving 3,791 asthma patients and 2,185 controls were analyzed [32] [33]. Our findings showed no significant relationship between the CC genotype of LTC4S -444 A>C polymorphism and susceptibility to asthma. Such a conclusion may be explained by differences of sample size, genotyping method, research design and statistical approach. Subgroup analyses were conducted based on ethnic and control group sources. Identically, no significant relationship was observed no matter in Asian or Caucasian population, nor population-based or hospital-based control group. Notably, subjects in control group could be healthy or accompanied with other diseases except for asthma which may influence the research quality. TSA reduces random errors caused by repeated measurements of inadequate data and provides a reliable conclusion through combining multiple relevant researches. Here, TSA was conducted to control the risk of type I errors and estimate the necessarily for further experiments. In our analysis, the cumulative zcurve did not cross the monitoring boundary, and the sample size was insufficient. Therefore, we strongly considered that the conclusion obtained from this analysis required for solid validation. In addition, asthma is a multifactorial disease. The pathogenesis of asthma is closely linked to the interaction of various genes and environmental factors, not a single gene. Therefore, the interaction between environmental factors and genetic variations is of significance in assessing genetic polymorphism. In the future research, more data are needed to take into consideration of gene polymorphism in influencing asthma.

## Conclusions

No significant correlation between the LTC4S -444 A>C polymorphism and susceptibility to asthma. Researches with high-quality and large sample size are required for further validation in multi-center hospital.

## Dodatak

### Conflict of interest statement

All the authors declare that they have no conflict of interest in this work.
